# Medinal as a Hypnotic

**Published:** 1910-02

**Authors:** 


### ABSTRACTS.

### Medinal as a Hypnotic

Dr. G. Likudi writing in the *Berliner klin. Wochenschrift* of November 8, 1909, from the Preparatory Therapeutic Clinic of Professor A. Fawitzki of the Imperial Military Academy of Medicine at St. Petersburg, reports his experience with medinal (mono-sodium salt of the di-ethyl-barbituric acid). The author welcomes the addition of medinal to the already long series of known hypnotics because it offers all the advantages of the di-ethyl-barbituric acid (veronal) (which he and other observers have up to now considered as coming nearest to a much desired hypnotic capable of producing nearly normal sleep), but adds free solubility, the lack of which has limited the general usefulness of the acid and frequently even made its administration impossible.

The author submits condensed histories of eleven cases treated at the clinic and the important data of fourteen cases from his private practice, in the course of which he administered medinal altogether eighty-six times, in the majority of cases per os, but several times also per rectum. Satisfactory results were almost always obtained with 7½ grains of medinal and only in four instances (habitues of narcotica and excessive nervous excitement) 15 grains had to be given. In order to exclude the possibility of psychic influence (suggestion), the drug was never made known to the patients as a hypnotic. At various occasions sugar was substituted on alternate evenings for medinal and the results carefully compared.

Amongst the cases of agrypnia treated at the clinic were: three cases of neurasthenia cerebrospinalis, one of tuberculosis pulmonum, two of pneumonia chron. (tbc.), two pleuritis, one of pericarditis exsudativa, one of convalescence from typhoid fever and one of arteriosclerosis with bronchitis chron. In the fourteen private cases the author dealt with: five cases of neurasthenia cerebrospinalis, two of hysteria, two of anemia (chlorosis), one of morphinism (trigeminal neuralgia with narcotica habit), two of erysipelas faciei with very high temperatures, and two of asthma bronchiale in which the author's results coincide with those of L. Ebstein, to the effect that the asthmatic attacks appear to be shortened and to become milder under the influence of medinal.

The author recommends further study of this interesting action of medinal in a larger number of cases, also of the apparent general sedative properties of the drug which have already been observed by Rabòw, Ebstein, and Pratoand of which he, too, has had favorable evidence.

Summarising, the author's condensed impressions are as follows:

1. Medinal is a rather reliable hypnotic; administered 86 times, it has failed to give satisfactory results in only 2 instances (Erysipelas faciei with temperatures above 102).

2. Its action is mild; sleep ensues on an average after thirty minutes and has in the majority of cases a sound, restful character, with ready normal awakening in the morning. After-effects in the form of dizziness, sensation of "heaviness in the head" and headache were observed in eight persons and after 32 per cent. of the total number of cases, but with the exception of one case (hysteria gravis with tendency to migraine) passed off two to three hours after rising.

3. The hypnotic action of medinal seems to be quicker in patients suffering from a general atony of the reacting organism, and slower in the presence of excessive nervous excitement, high temperatures and severe painful coughing spells. The average duration of sleep under the influence of medinal is from seven to eight hours with occasional short interruptions in the presence of cough or nervous irritation. It can be prolonged by the combination of medinal with other suitable narcotica.

4. In addition to its soporific action medinal seems to exercise a sedative influence on the nervous system and to render asthmatic attacks shorter and milder.

5. The average effective dose of medinal is 7½ grains; only in exceptional cases 15 grains are required. The drug is tolerated equally well when administered per os (absence of dyspeptic effects or per rectum (non-irritant).

In the *Muenchener Med. Wochenschr.* of October 12, 1909, Dr. E. Steinitz writing from Professor Rostoski's Division of the Stadtkrankenhaus Friedrichstadt in Dresden (Germany), confirms his previously reported favorable results with medinal. The author's first article appeared in *Therapie der Gegenwart*, July, 1908. He particularly emphasises the prompt and rarely failing efficiency of medinal when administered per rectum and recommends for this purpose the use of medinal suppositories. Surprisingly good results were obtained with medinal in this form in asthma bronchiale and stenocardia, the nocturnal attacks being shortened and frequently entirely forestalled by its use, although this particular action of medinal seemed to decrease after long and continuous administration and to necessitate gradual increase of the doses. Generally, medinal suppositories will be indicated wherever small doses are desired and stomachal hypersensitiveness is to be reckoned with. The combination of medinal with morphine did not only prove useful in certain cases, but, according to extensive experiments of an American physician, medinal appears to effectually counteract the disagreeable side effects of morphine. Neither from his own nor from the observation of others does the author feel justified in drawing definite conclusions regarding medinal when administered subcutaneously and recommends further experiments with 15 grains in a 10 per cent solution.

The author then reviews a number of other contributions to the therapy of medinal, the great majority of which concur in recognising the superiority of medinal over di-ethyl-barbituric acid (Munck), who has administered it, mostly subcutaneously, in conditions of excitement of the insane, dissents somewhat, but admits the more agreeable taste of the medinal solutions. Only when placed directly on the tongue, medinal tastes strongly bitter, owing to its far greater solubility as compared with the di-ethyl-barbituric acid. The author recommends that hospitals and sanitaria keep stock solutions of medinal (56 grains in ten ounces of water, one dessertspoon of which contains 1½ grains). Addition of more water, or wine, flavors, morphine, codeine, and the like, if desired, should be made to the individual doses, not to the stock solution, to avoid precipitates. The author has also experimented with the mono-sodium salt of the di-propyl-barbituric acid (solubility in water 1:3) which produces sleep on an average in ten minutes, its other characteristics being identical with those of medinal. It is, however, not possible, as was hoped, to obtain permanent satisfactory hypnotic action with this salt by smaller doses than those necessary of medinal. The author does not attribute practical therapeutic importance to either the di-propyl-barbituric acid or its mono-sodium salt.

---

STORIES about fattening oysters in polluted fresh-water beds, and shipment in unsanitary receptacles have caused many people to look with suspicion upon the bivalve. They needn't worry any more. Uncle Sam has decided that such practises are violations of the food and drug act of June 30, 1906, in that the shellfish contain an added "poisonous or other added deleterious ingredient which may render such articles injurious to health."

